# Determinants and Suitability of Carotenoid Reflection Score as a Measure of Carotenoid Status

**DOI:** 10.3390/nu12010113

**Published:** 2020-01-01

**Authors:** Elaine Rush, Isaac Amoah, Tung Diep, Shabnam Jalili-Moghaddam

**Affiliations:** 1 Faculty of Health and Environmental Studies, Auckland University of Technology, Auckland 1142, New Zealand; isaac.amoah@aut.ac.nz (I.A.); tung.diep@aut.ac.nz (T.D.); shabnam.jalili@aut.ac.nz (S.J.-M.); 2 Riddet Centre of Research Excellence, Palmerston North 0632, New Zealand

**Keywords:** vegetable and fruit intake, carotenoids, biomarker, Veggie Meter™

## Abstract

Carotenoids, orange-coloured pigments found in vegetables, fruit, eggs and dairy foods, act as antioxidants and vitamin A precursors in the human body. Skin carotenoid concentration is a biomarker of vegetable and fruit intake. The aim was to identify determinants of skin carotenoid concentration by measuring “Veggie Meter™” carotenoid reflection spectroscopy scores (CRS) from the fingertip of adults with a range of ages, ethnicity and body size. Frequencies of daily intake of vegetables and fruit and weekly intake of pumpkin and carrot, dark green leafy vegetables (DGLV), eggs (yolk), and dairy were determined from a self-completed food-frequency-questionnaire. A total of 571 (324 Women, 247 Men) adults, aged 16 to 85 years, completed measurements. The CRS ranged from 83 to 769, with a median of 327. Women and men did not score differently. For all participants there were negative correlations of CRS with weight (*r* = −0.312) and BMI (*r* = −0.338) and positive correlations with weekly intakes of DGLV (*r* = 0.242) and carrots and pumpkin (*r* = 0.202). Based on a review of health outcomes associated with plasma carotenoids, 82% of the participants in the current study are at moderate risk, or more, of negative health outcomes. Determinants of carotenoid status were body size, intake of DGLV, carrots and pumpkin, and ethnicity.

## 1. Introduction

A recent systematic review and meta-analysis [1] of 95 prospective studies has established a strong association between the reported consumption of vegetables and fruits with reduced incidence of chronic diseases, including diabetes, hypertension, coronary heart diseases and cancers. Globally, food-based dietary guidelines [2] encourage an increase in the quantity and variety of vegetables and fruit consumed because they are micronutrient-dense, rich in fibre and contain many phytochemicals with health-promoting bioactivity. The consumption of more vegetables than fruits is encouraged. The measurement errors in self-reports of dietary intakes, including fruits and vegetables, are additive and it is important to validate reports with the use of biomarkers [3].

Carotenoids, including beta carotene, alpha carotene, lycopene, lutein and xanthophylls are orange pigments ubiquitous in vegetables. Fruits [4] and some animal products such as eggs and dairy contribute provitamin A, antioxidant and chemoprotective components to the diet. Carotenoids are found mainly in vegetables [5,6]. For example, carrot, kale, spinach and pumpkin are good sources of beta carotene, corn contains lutein, and tomato is a good source of lycopene [7].

In New Zealand there is the ability to grow and market vegetables and fruits throughout the year. Yet, in the annual health survey only one third of respondents report that they consume 3 or more servings of vegetables plus 2 or more servings of fruit each day [8]. In addition, the same survey based on measures of height and weight records that the prevalence in adults of obesity and overweight is 34% and 31% respectively. Overweight and obesity are forms of malnutrition and may coexist with micronutrient deficiencies. While the frequency of vegetable and fruit intake is subjectively assessed by the Ministry of Health every year, these reports of vegetable and fruit intake have not been validated with a biomarker.

Concentrations of carotenoids in plasma are well established as biomarkers of vegetable and fruit intake [9,10,11]. Noninvasive point-of-care determination of carotenoid concentration is of benefit to individuals and health professionals. Advantages are convenience, less expense and time and the immediate opportunity to discuss the measurement with the individual. One such test for carotenoids is a measure of skin colour, also shown to be a valid and reliable biomarker for vegetable and fruit intakes [12,13]. Skin colour measurement by reflection spectroscopy is the principle of the “Veggie Meter™”, a relatively small, portable instrument that interfaces with a dedicated laptop computer. The tip of the finger is pressed lightly over the head of a white light probe to momentarily remove the blood from the finger-tip, and the reflected light is then measured. This relatively rapid measurement, less than 15 s, enables immediate feedback and interpretation of the measurement to the individual. The device has been used to evaluate carotenoid reflection scores (CRS) of participants in the United States of America [14] and Japan [15] but not in New Zealand.

This study aimed to profile and identify determinants of fingertip CRS in women and men of five ethnic groups with a range of age, body size and self-reported intake of foods including vegetables and fruits containing carotenoids.

## 2. Materials and Methods

### 2.1. Setting and Participants

This cross-sectional study of members of diverse community groups selected to represent a convenience sample that varied by age, ethnicity and socioeconomic status determined by geographic location. For a prediction equation with three predictors and based on a moderate effect size of 0.15, a power of 0.80, and a probability level of 0.05, it was determined that the minimum sample size would be 76. Thirteen sites in Auckland and Hamilton, New Zealand responded to an invitation to take part. In all, 44% of participants were from 9 workplaces, 39% staff and students from the three campuses of the Auckland University of Technology and 17% from community groups such as Rotary Clubs. The Auckland University of Technology Ethics Committee provided ethical approval 18/420. Inclusion criteria were that participants were aged 16 years or more and able to give informed consent. Intentionally, to increase the diversity of the participants there were no further exclusion criteria. The main benefit to the participants was an immediate measurement of carotenoid status and general education about vegetable and fruit consumption. A total of 571 participants were measured by researchers with expertise in nutrition. All participants provided informed signed consent before taking part. Age, gender, smoking status, self-identified ethnic group and their self-reported height and weight were provided on a one-page questionnaire which also included questions about diet and supplement use.

### 2.2. Measurement of Carotenoid-Containing Food Intake

A 6-item food frequency questionnaire asked two questions from the annual national health survey about how many servings of vegetables and fruit were eaten in one day [16]. Daily servings of vegetables and fruit were summed. Specific questions concerning how many servings of carrot and pumpkin, dark green leafy vegetables, eggs (yolk) and dairy foods were eaten in one week were asked. These specific foods were selected because they are the most frequently consumed foods in the New Zealand diet that are significant sources of carotenoids [7].

### 2.3. Measurement of Skin Carotenoid Concentration

Pressure-mediated “Veggie Meter™” CRS (Longevity Link Corporation, Salt Lake City, UT, USA) were determined. The various carotenoid compounds—beta-carotene, lycopene, lutein, zeaxanthin, beta-crytoxanthin—and all isomers of these compounds generate a very similar yellow coloration that can be detected and quantified by the Veggie Meter. The manufacturer instructions were followed and the surface of the contact lens was regularly cleaned with an optical cloth. The instrument was calibrated prior to any measurements and every hour using the dark and white reference wedges provided by the manufacturer. After firmly wiping the fat pad at the tip of the right-hand index finger with a water-moistened swab, the finger-tip was dried and inserted into the finger port so that the tip was pressed over the contact lens. The finger was held gently in place by a spring-loaded clip. The CRS was recorded as the average of three consecutive measurements for each participant. The finger was removed from the port between measurements to allow reperfusion. If the average score was less than 250 or there was any irregularity, the measurements were repeated.

In a separate study, from 10 participants on three different occasions, blood was sampled at the same time as the CRS measurement, with the same Veggie Meter used for this study. Plasma carotenoids were measured by spectroscopy (Cary 400, Agilent Technologies, Victoria, Australia) at the Christchurch Health Laboratories (CHL). They reported plasma carotenoid concentration in µmol/L; laboratory reference range 1.5 to 3.0 µmol/L. Regression of the CRS against the plasma measure resulted in the equation: CHL concentration µmol/L = 0.0055 × CRS + 0.0197, SEE = 0.439 *r*^2^ = 0.72. The equivalent reference range for the CRS was therefore 286 to 480. However, given the relatively wide 95% CI of the mean at these values, the “healthy” reference range for communication with the participants was set at 250 to 530 and we termed this a screening test. In other words, if the CRS was less than 250, we actively encouraged consumption of more vegetables and fruit, and if more than 530 we endorsed that they were eating, and should continue to eat, plenty of carotenoid-containing foods.

### 2.4. Statistical Analysis

Categorical variables are reported as count and prevalence (%). Where more than one ethnic group was identified by a participant, ethnic group was prioritized in the order Maori, Pacific, Asian (China and South East Asia); South Asian, European and other (Middle Eastern, African, Latin American). Continuous variables were checked for normality and are reported as mean, standard deviation and range. Comparisons by gender, ethnicity and age were assessed by unpaired t-test and ANOVA. Correlation was determined by visual inspection of scatter plots and Pearson *r*. Determinants of the CRS, previously identified by simple correlation, were entered stepwise, forward and backward into multiple regression to find the best combination of predictors. The absence of collinearity was confirmed by Variance Inflation Factors < 2. A regression equation was derived where CRS = β1 × determinant1 + β2 × determinant2 + β3 × determinant3. The β coefficients are the degree of change of CRS for one unit of change for the variable. The change in *R*^2^, the multiple correlation coefficient of determination, was used to determine how much variance in the dependent variable (CRS) was accounted for with the addition of each determinant. Analyses were performed with IBM SPSS statistics software (version 25.0, IBM Corp., Armonk, New York, NY, USA).

## 3. Results

### 3.1. Participant Characteristics and Carotenoid Reflection Scores

In total, 571 participants were measured—324 women and 247 men. Of these, 6.7% self-identified their priority ethnicity as Maori, 14.7% as Pacific, 17.3% as Asian, 12.6% as South Asian, 41.0% as European, and 7.7% as other (Middle Eastern, African, Latin American). The average age was 39 ± 17 (mean ± SD, range 16 to 85) years; weight 76 ± 20 (range 41 to 170) kg; height 170 ± 10 (range 140 to 200) cm; and body mass index (BMI) 26 ± 6 (range 15 to 59) kg·m^−2^. Twenty-seven participants did not provide their height or weight due to an omission in the questionnaire. These participants were European, 13 years older and had higher scores (60 units) than those that provided weight and height. Seven percent (40/571) of the participants reported that they currently smoked tobacco.

**Figure 1:**
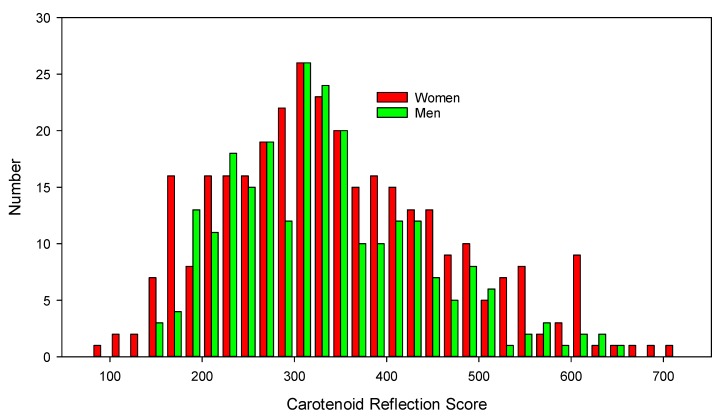
Histogram of carotenoid reflection scores in the study population.

Applying the cutoffs based on the screening test criteria, one out of five (21.4%) had a low CRS (<250), one out of three (32.2%) <280, with one out of 14 (7.1%) scoring higher that than 530 (Figure 1). Older participants (>40 years) had CRS 21 units higher than younger participants (95% CI 40, *p* = 0.033) (Table 1). By ethnicity, Pacific CRS were significantly lower than all other groups and Asian significantly higher than all groups except for “other”. There were no differences by gender within ethnic groups (Table 1).

### 3.2. Carotenoid Food Intake

**Figure 2:**
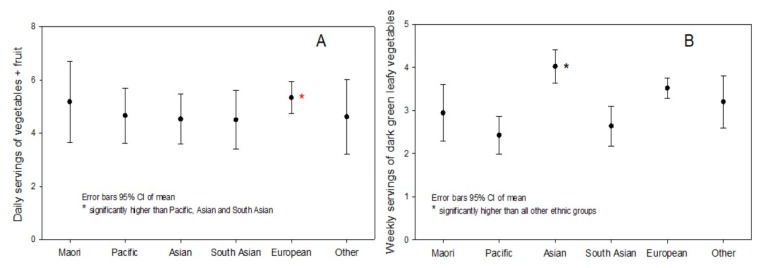
Comparison by ethnicity of (**A**) daily vegetable and fruit intake and (**B**) weekly dark green leafy vegetable intake.

Slightly more than half (53.3%) reported consuming 3 or more servings of vegetables a day and 61.6% 2 or more fruit a day, with 39.4% reporting 3 or more vegetables plus two or more fruit a day (i.e., 5+ a day). Women reported consuming around 0.5 servings a day of vegetables, and 0.5 servings of dark green leafy vegetables weekly more than men (Table 2). By ethnicity, Europeans reported almost one more serving a day of fruit plus vegetables than Pacific, Asian and South Asian but were not different to Maori or Other ethnic groups (*p* = 0.015, ANOVA) (Figure 2A). Weekly consumption of dark green leafy vegetables was reported as 4.0 ± 2.0 by Asian which was 0.5, 0.8, 1.4, 1.1 and 1.6 servings higher than European, “Other”, Maori, South Asian and Pacific respectively (Figure 2B).

### 3.3. Associations between Carotenoid Reflection Score and Determinants

For all participants there were moderate to strong negative correlations of CRS with weight and BMI and positive correlations with age, vegetables, fruit and vegetables plus fruit intakes and weekly intake of dark green leafy vegetables and carrots and pumpkin (Table 3). Fruit (but not vegetables) were a determinant for men but not women and carrots and pumpkin were a determinant for men but not women.

### 3.4. Predictors of Carotenoid Reflection Score

Based on the statistically significant correlations in Table 3, determinants of CRS were examined systematically by stepwise regression. Body weight and vegetable intake as predictors were excluded from the model as they exhibited collinearity with BMI and dark green leafy vegetable intakes respectively. There was overlap in the general questions about vegetables and fruits and the specific questions concerning dark green leafy vegetables and carrots and pumpkin, so vegetable and fruit intakes was were excluded from the model. The best model had four predictors that explained a total of 16.2% of the variation (Table 4). Body mass index explained 10.9% of the variation with each unit of increase of BMI associated with a reduction in the CRS of 5.4 units. Weekly intake of dark green leafy vegetables explained an additional 2.9%, with 1 serving a week adding 6.7 units to the CRS. One serving a week of carrots or pumpkin added 9.4 units to the CRS. Asian ethnicity added 17 units. Therefore, determinants of an increased CRS were a smaller body size, weekly intake of dark green leafy vegetables, carrots and pumpkin and Asian ethnicity.

While smoking tobacco was not a predictor in the above model, there was a difference between smoking or not. Smokers’ mean CRS was 274 ± 116 (*n* = 40) and non-smokers 346 ± 115 (*n* = 531). On average, smokers were 72 score units (95% CI 34, 110), *p* < 0.001) lower than non-smokers.

Of the 571 participants there were 10 couples living in the same house, for 7/10 the man had a higher score but overall this was not significantly different.

## 4. Discussion

To our knowledge, this is the first relatively large, multi-ethnic study to measure and validate carotenoid status using Veggie Meter™ carotenoid reflection scores. The most important finding is that body size, measured both as weight and BMI, was the strongest determinant of carotenoid status. We also show a relatively high prevalence of low carotenoid status, with no overall difference between women and men that decreases with age. The highest scores for Asian and lowest for Pacific are of importance alongside the observation that Asian participants reported the highest intake of green leafy vegetables and Pacific the lowest. Each of these findings will be discussed in turn.

Very few studies have used the Veggie Meter™ to assess CRS. Only one study in Japan [15], with 985 participants, mean age 69 years, has reported a negative correlation (*r* = −0.087) of CRS with BMI, a surrogate measure of body fatness, over a similar range to that in this study. We add to evidence for a negative association with BMI and the strength of our association was higher (*r* = −0.338) for all participants, and was present for women and men when analysed separately. Height was not correlated with CRS. Explanations for this association with body composition include that carotenoids are fat soluble and the concentration in body tissues will depend on both the amount (dose) ingested and absorbed [17] and also the volume of distribution. Larger people will have more absolute fat mass and higher body fat percentage. Also, excess body weight is associated with inflammation, which may deplete carotenoid stores [18] through oxidation. Finally, while servings were not quantified, a person weighing 100 kg with the same percentage body fat as a person weighing 50 kg will require twice the dose of carotenoids to achieve the same tissue concentration. No allowance for body size is made with generalized vegetable and fruit guidelines, yet we agree with others [19] that carotenoid requirements may be dependent on body composition.

It was not surprising that CRS scores of this study population were relatively low considering the low reported intake of vegetables and fruit in this study. Only 39.2% met the guideline of both 3+ vegetables and 2+ fruit a day. This is the same as the 2018, 2019 National Health Survey [8] which reports 39.4% of the New Zealand population meeting the guideline. The same survey shows that men eat less vegetables and fruit than women, with higher intakes of vegetables for Maori and European than Pacific and Asian ethnicities. However, the Ministry includes South Asian within the group Asian. We show a difference in diet between Asian and South Asian reflecting known differences in dietary patterns [20], otherwise our findings for vegetable and fruit intake are similar to the national survey.

Compared with the small number of other studies that have used the Veggie Meter™ technique, our mean CRS value of 346 is higher than that recorded in a diverse community sample in Eastern North Carolina USA of ~240 in African American and Non-African American (*n* = 469) [21] and 57 from an eye clinic in Utah, USA [14] with mean CRS of 297, but similar to a Japanese study where the mean CRS was 342 [15]. While we show no significant difference in CRS between men and women, the Japanese [15] study demonstrated that Japanese women scored higher than men (382 vs. 315).

A review of mostly prospective studies of health outcomes in relation to plasma carotenoids proposed a carotenoid health-based index for significant positive health outcomes including metabolic syndrome and cancer. Derived from our validation equation, the equivalent CRS score for their cut-point criteria of very high risk of <1 µM is 180, high risk 1–1.5 µM is 270, moderate risk 1.5–2.5 µM is 450, low risk 2.5–4 µM is 730. This means that 82% of the participants in the current study are at moderate risk or more of negative health outcomes. This lends support to strengthening the vegetable and fruit guideline to more than 3 vegetables a day and to encourage increased intake of a variety of dark green leafy and orange vegetables.

The difference in CRS observed by age may be related to higher socioeconomic status for the older people and therefore an ability to afford to purchase or grow greater quantities and more diverse vegetables and fruit and also time and ability to cook and prepare meals. Older participants were also more likely to be European.

Women reported that they consumed more vegetables and more dark green leafy vegetables than men. Women and men reported the same intake of carrots and pumpkins but only in men was intake significantly correlated with the CRS. This may relate to differences, by gender, in dietary recall, over and under-reporting and body size. The collinearity of the dietary questions infers that those who report that they eat more vegetables also report that they eat more fruit and within vegetables more carrot, pumpkin and dark green leafy vegetables are reported. In the multiple regression, gender was not a significant predictor. Another explanation is that women, in general, have a higher proportion of body fat than men. Carotenoids are fat soluble and therefore the same intake would result in more dilution in the fat for women than for men. In future studies it would be important to have a measure of body fat and to explore a gender difference in dose–response further.

A major omission of the dietary questions was not asking a question about tomato consumption, which would be a measure of the carotenoid lycopene. On the other hand, we did show that reported vegetable intake, dark green leafy vegetables and orange vegetables (carrot and pumpkin) was positively associated with CRS. We also did not formally ask about or record any digestive problems which could impair absorption of carotenoids because this was not part of the ethical approval. The representativeness of the study population was limited. Strengths of this work are the wide age range, diverse settings and the comparison of the five main ethnic groups living in Auckland, New Zealand. We also demonstrated the feasibility of using the Veggie Meter in the workplace at the same time as providing encouragement for the individual to increase intake and for the employer to support this.

## 5. Conclusions

Future investigations should include targeted interventions, children, members of the same household, a more objective measure of body fat, include tomatoes in the questionnaire and ask about digestive problems.

A considerable body of evidence exists that supports the contention that a diverse variety of and more vegetables should be consumed every day for health. The quantity should be in proportion to body size. Nationally, New Zealand, as a primary producer, exports a large quantity of premium quality fruit and vegetables [22], which implies that there is not a shortage of supply. This important public health issue has long term implications for prevention of non-communicable diseases and calls for action to improve access to vegetables and fruit. One way this could be done is to remove the goods and services tax from vegetables and fruit. Food-based dietary guidelines need to emphasise the importance of the diversity of vegetables and fruits required and consider serving size guidelines that take into account that larger people should consume a greater volume of vegetables than smaller individuals. Vegetable and fruit preferences of men and women and by ethnic group differ and dietary advice to improve carotenoid status should be tailored to gender and ethnicity.

Determinants of carotenoid status were body size, intake of DGLV, carrots and pumpkin, and ethnicity.

## Figures and Tables

**Table 1:** Carotenoid reflectance scores of participants by age, ethnicity and gender.

	Carotenoid Reflection Scores				
Characteristic	*N* (Women, Men)	All	Women	Men	Difference Women − Men (95% CI)
All	571 (324, 247)	342 ± 116	345 ± 124	336 ± 105	10 (−9, 29)
Age ≤ 40 years	334 (201, 133)	333 ± 119	338 ± 122	325 ± 114	13 (−13, 39)
>40 years	237 (125, 122)	354 ± 111 ^1^	358 ± 125	349 ± 93	9 (−20, 37)
Ethnicity					
Māori	38 (25, 13)	315 ± 110	293 ± 117	360 ± 83	−66 (−140, 8)
Pacific	84 (42, 42)	257 ± 85 ^2^	247 ± 84	267 ± 87	−20 (−58, 17)
Asian	99 (62, 37)	388 ± 115	400 ± 119	367 ± 105	32 (−14, 80)
South Asian	72 (37, 35)	341 ± 113	340 ± 110	343 ± 118	−3 (−58, 50)
European	234 (137, 97)	352 ± 111	359 ± 115	341 ± 112	17 (−11, 46)
Other	44 (23, 21)	367 ± 126	367 ± 157	367 ± 84	0 (−78, 78)

*N* number, mean ± SD carotenoid reflection score, ^1^ *p* < 0.03 for difference by age independent *t* test. ^2^ *p* < 0.001 by ethnicity ANOVA.

**Table 2:** Reported number of servings consumed each day or week of foods containing carotenoids.

Food Frequency	All (Range)	Women (Range)	Men (Range)	Difference (95% CI)	*p* ^1^
Vegetables/day	2.8 ± 1.4 (0,7)	3.0 ± 1.6 (0,7)	2.5 ± 1.3 (0,6)	0.5 (0.3, 0.8)	<0.001
Fruit/day	2.1 ± 1.4 (0,7)	2.2 ± 1.4 (0,7)	2.0 ± 1.3 (0,7)	0.2 (−0.1, 0.4)	0.181
Fruit + vegetables/day	4.9 ± 2.4 (0,14)	5.2 ± 2.5 (0,14)	4.6 ± 2.2 (0,11)	0.7 (0.3, 1.0)	0.001
Dark green leafy vegetables/week	3.2 ± 2.8 (0,7)	3.5 ± 2.0 (0,7)	3.0 ± 2.0 (0,7)	0.5 (0.2, 0.8)	0.003
Carrots/pumpkin/week	2.7 ± 1.8 (0,7)	2.7 ± 1.7 (0,7)	2.6 ± 1.8 (0,7)	0.0 (−0.3, 3.0)	0.770
Eggs/week	2.6 ± 1.8 (0,7)	2.4 ± 2.4 (0,7)	2.8 ± 2.5 (0,7)	−0.4 (−0.8, 0.0)	0.078
Dairy/week	4.6 ± 2.2 (0,7)	4.5 ± 2.3 (0,7)	4.7 ± 2.2 (0,7)	−0.2 (−0.6, 0.2)	0.345

Mean ± SD, ^1^ Independent *t* test equal variances not assumed.

**Table 3:** Correlation of carotenoid reflection scores with participant characteristics and diet.

Pearson Correlation Coefficient, *r* (95% CI)			
Determinant	All	Women	Men
Age	0.146 (0.065, 0.225) ***	0.164 (0.056, 0.268) **	0.130 (0.005, 0.251)
Weight	−0.312 (−0.384, −0.236) ***	−0.397 (−0.485, −0.301) ***	−0.251 (−0.364, −0.130) ***
Height	−0.078 (−0.159, 0.004)	−0.078 (−0.185, 0.031)	−0.067 (−0.190, 0.058)
BMI	−0.338 (−0.409, −0.263) ***	−0.393 (−0.481, −0.297) ***	−0.248 (−0.362, −0.127) ***
Vegetables/day	0.231 (0.152, 0.307) ***	0.273 (0.169, 0.371) ***	0.142 (0.017, 0.262) *
Fruits/day	0.169 (0.088, 0.248) ***	0.089 (−0.020, 0.196)	0.303 (0.185, 0.412) ***
Vegetables plus fruit/ day	0.243 (0.164, 0.319) ***	0.227 (0.121, 0.328) ***	0.265 (0.145, 0.377) ***
Dark green leafy veg/week	0.242 (0.187, 0.340) ***	0.266 (0.162, 0.364) ***	0.199 (0.076, 0.316) **
Carrots/pumpkin/week	0.203 (0.123, 0.280) ***	0.138 (0.029, 0.243)	0.302 (0.184, 0.411) ***
Eggs/week	0.067 (−0.015, 0.148)	0.054 (−0.055, 0.162)	0.096 (−0.029, 0.218)
Dairy/week	0.055 (−0.027, 0.136)	0.024 (−0.085, 0.133)	0.112 (−0.013, 0.234)

* *p* < 0.05, ** *p* < 0.01 and *** *p* < 0.001 denote statistically significant difference.

**Table 4:** Multiple regression analysis with carotenoid reflection score as the independent variable.

Independent Variable	Regression Coefficient, β	SE, β	^a^*R*^2^ (P)
Constant	439.660	24.940	(<0.001)
BMI, kg/m^2^	−5.440	0.878	0.109 (<0.001)
Dark green leafy vegetables/week	6.670	2.504	0.138 (<0.001)
Carrots/pumpkin/week	9.380	2.738	0.154 (0.001)
Ethnic group ^b^	16.981	7.029	0.162 (0.016)

^a^ Adjusted for the number of predictors. ^b^ Ethnic group codes as European 0, Asian 1 and all other groups −1.
